# Sol–gel synthesis of Eu^3+^ doped silica-gold nanorod composites with tunable optical properties†

**DOI:** 10.1039/d3ra04652d

**Published:** 2023-09-08

**Authors:** José Raúl Montes-Bojorquez, Ofelia Hernández-Negrete, Hilda E. Esparza-Ponce, Víctor Alvarez-Montaño, Javier Hernández-Paredes

**Affiliations:** a Departamento de Física, Universidad de Sonora (UNISON), Edificio 3F, Blvd. Luis Encinas J. y Rosales S/N Col. Centro Hermosillo Sonora C.P, 83000 Mexico jahernandezparedes@gmail.com; b MEMS Research Lab, Department of Physics and Astronomy, University of Texas at San Antonio 1 UTSA Circle San Antonio TX 78249 USA; c Departamento de Ingeniería Química y Metalurgia, Universidad de Sonora (UNISON), Edificio 5I, Blvd. Luis Encinas J. y Rosales s/n Col. Centro Hermosillo Sonora C.P, 83000 Mexico ochernandeznegrete@gmail.com; d Centro de Investigación en Materiales Avanzados S. C. (CIMAV) Miguel de Cervantes 120, Chihuahua Chihuahua C.P, 31136 Mexico

## Abstract

Gold nanorods (AuNRs) suspension at various concentrations was added into the sol–gel process to engineer nanostructured europium-doped silica host matrices as light-emitting composites. For this purpose, the samples were prepared following two different routes depending on the chemicals used as dopant and catalyst: (a) Eu(NO_3_)_3_·5H_2_O and HNO_3_, and (b) EuCl·6H_2_O and HCl. In any case, samples adding various concentrations of AuNRs suspension were prepared. The structural characterization of the samples was through STEM, backscattered electrons (BSE), and EDS analysis. Additionally, their optical properties were evaluated by PL spectroscopy and CIE colorimetry. The results confirmed that (a) methodology produced samples with AuNRs embedded and randomly distributed in the samples. However, these features were not observed in the samples obtained through (b) due to AuNRs dissolution in HCl media. Regarding the optical properties, the analysis of the relative intensity ratio ^5^D_0_ → ^7^F_2_/^5^D_0_ → ^7^F_1_ suggested that Eu^3+^ ions occupy non-centrosymmetric sites in (a) host matrices and centrosymmetric sites in (b). Hence, the increase of AuNRs suspension when fabricating (a) host matrices produced remarkable color changes in the luminescence of the samples towards the reddish-orange region. Meanwhile, the dissolution of AuNRs in (b) minimized the localized surface plasmon resonance (LSPR) effects on the Eu^3+^ luminescence. These findings revealed that the evaluation and selection of chemicals are critical factors when engineering these materials for more efficient coupling between the LSPR and Eu^3+^ luminescence.

## Abstract 2

The selection of chemicals was crucial for AuNRs stabilization in europium-doped silica host matrices with tunable optical properties.

## Graphical abstract

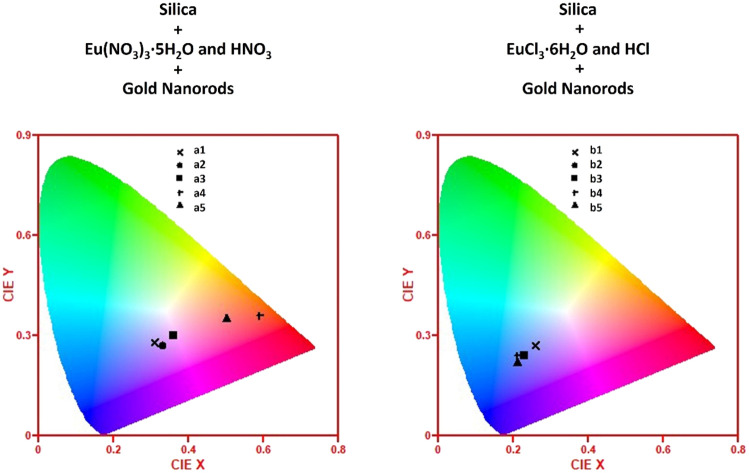

## Introduction

The sol–gel technique is a versatile route for the synthesis of materials with diverse morphological characteristics.^1,2^ For example, this technique is often used to produce low-cost amorphous silica in different shapes, such as blocks, films, and nanostructures, for various applications in science and technology.^3–5^

Silica is a chemical compound that has attracted the attention of scientists and technologists as a result of its relatively low toxicity, minimal ecological impact, and low production costs.^6,7^ Therefore, silica is a suitable host matrix for diverse components such as atoms, ions, molecules, nanostructures, and particles.^8^ In this regard, the confinement of rare earth (RE) ions in silica hosts is a logical approach for engineering materials with luminescent properties because silica hosts can be easily fabricated in various shapes and sizes for their practical use.^9–11^ Furthermore, the overall optical response of the RE ions can be enhanced by altering their local environment with structural arrangements in the host matrix to produce luminescent materials with applications in lighting, laser technologies, optical imaging, and medicine, to give some examples.^12,13^

Among the RE ions, the Eu^3+^ shows distinctive luminescent bands in the visible region of the electromagnetic spectrum upon excitation with UV light. This feature can potentially be exploited to give characteristic red luminescence to materials.^14^ Besides, the optical response of Eu^3+^ can be dramatically changed because of its hypersensitive ^5^D_0_ → ^7^F_2_ transition, which is significantly affected by the local symmetry.^14^ Therefore, the contribution of the Eu^3+^ to the overall luminescence can be tuned by making structural changes in the silica host.

Likewise, it is known that noble metal nanoparticles, *e.g.*, gold nanoparticles (AuNPs) or silver nanoparticles (AgNPs), have interesting optical properties as a result of their LSPR.^15^ According to the literature, when a nanoparticle shows LSPR, the intensity of the electromagnetic field can be enhanced up to several times in its proximity.^16^ Therefore, it is not surprising that this phenomenon can be further exploited for tuning the optical properties of diverse materials.^17^ Hence, some studies support using noble metal NPs to enhance the RE ions' luminescent properties.^16^

Herein, we report the addition of AuNRs suspension at various concentrations into the sol–gel process to obtain nanostructured composites based on europium-doped silica host matrices (SiO_2_:Eu^3+^). For this purpose, the methodology consisted of preparing a separate AuNRs suspension using the approach depicted by El Sayed.^18^ Then, AuNRs were incorporated into the sol–gel process to obtain the desirable nanostructured materials. For the sol–gel process, two different routes of synthesis were evaluated. The differences between these synthesis routes consisted of the chemicals used as dopants and catalysts: (a) Eu(NO_3_)_3_·5H_2_O and HNO_3_, and (b) EuCl·6H_2_O and HCl.

In the presence of acid catalysts, silicon alkoxides form dense and transparent silica gels with small pore sizes.^19^ In the literature on sol–gel, the reason for selecting a given acid is rarely explained. In contrast, the differences in the type of acid on the morphology and microstructure characteristics reported elsewhere^20,21^ have been attributed to the nucleophilic nature of each anion.^22^

In this work, nitric and hydrochloric acids were chosen as common catalysts along with europium nitrate and europium chloride, respectively, to minimize chemical complexity. In any case, samples adding various concentrations of AuNRs suspension were prepared. This methodology produced two series of composite materials structurally characterized using SEM, Backscattered Electrons BSE, and EDS analysis. Additionally, their optical properties were evaluated by PL spectroscopy and CIE colorimetry.

## Materials and methods

### Materials

AuNRs suspension was prepared with the following chemicals: hexadecyltrimethylammonium bromide CTAB (≥96%), benzyldimethylammonium chloride dihydrate BDAC (98%), sodium borohydride NaBH_4_ (99%), l-ascorbic acid (99%), HAuCl_4_·3H_2_O (≥49.0% Au basis), AgNO_3_ (≥99%) purchased from Sigma-Aldrich Mexico. Deionized water (18 MΩ) was used for these experiments.

Europium-doped silica matrix with and without AuNRs were prepared with the following chemicals: tetraethoxysilane TEOS (98%), ethanol (99.5%), hydrochloric acid HCl (33–40%), nitric acid HNO_3_ (65–67%), europium(iii)chloride hexahydrate EuCl·6H_2_O (99.99%), europium(iii)nitrate hydrate Eu(NO_3_)_3_·5H_2_O (99.9%) purchased from Sigma-Aldrich Mexico. Deionized water (18 MΩ) was used for these experiments.

### Synthesis

The preparation of the samples was carried out in two stages. First, the synthesis of AuNRs. Next, the synthesis of europium doped silica host matrices (a) and (b) adding various concentrations of AuNRs suspension (see Table 1). Pure silica host matrices were also prepared.

Samples prepared *via* sol–gel

**Table:** 

Sample	TEOS (mL)	Water (mL)	EtOH (mL)	Eu(NO_3_)_3_·5H_2_O 0.0597 M (mL)	HNO_3_ (μL)	AuNRs suspension (μL)
a0	0.60	0.45	1.95	—	150	—
a1	0.60	—	1.95	0.45	150	—
a2	0.60	—	1.95	0.45	150	100
a3	0.60	—	1.95	0.45	150	200
a4	0.60	—	1.95	0.45	150	400
a5	0.60	—	1.95	0.45	150	800

**Table:** 

Sample	TEOS (mL)	Water (mL)	EtOH (mL)	EuCl·6H_2_O 0.0597 M (mL)	HCl (μL)	AuNRs suspension (μL)
b0	0.60	0.45	1.95	—	150	—
b1	0.60	—	1.95	0.45	150	—
b2	0.60	—	1.95	0.45	150	100
b3	0.60	—	1.95	0.45	150	200
b4	0.60	—	1.95	0.45	150	400
b5	0.60	—	1.95	0.45	150	800

#### Synthesis of AuNRs

For the synthesis of AuNRs, we followed the methodology proposed by El-Sayed.^18^ This methodology is a two-step process.

Firstly, a seed solution was prepared by mixing CTAB (5 mL, 0.20 M) with HAuCl_4_ (5 mL, 0.50 mM). Then, 0.60 mL of freshly prepared ice-cold NaBH_4_ (10 mM) solution was added under vigorous stirring. The resulting brownish seed solution was stirred for an additional 5 min and stored at 25 °C for 30 min to achieve complete decomposition of the remaining borohydride ions. According to the literature, the rapid reduction of HAuCl_4_ by NaBH_4_ yields the *in situ* formation of CTAB-capped AuNPs with an average diameter of a few nanometers.^18^

Next, a growth solution was prepared separately by mixing CTAB (5 mL, 0.20 M), AgNO_3_ (50 μL, 4 mM) and HAuCl_4_ (5 mL, 1 mM). After gently stirring the above solution, 80 μL of ascorbic acid (0.0788 M) was added to reduce Au^3+^ to Au^1+^, causing the color of the growth solution to change from dark yellow to colorless. Finally, 12 μL of seed solution was added to the growth solution. Upon addition of the seeds, Au^1+^ further reduces to Au^0^, inducing Au deposition onto the surface of the seed nanoparticles. The temperature of the experiment was controlled between 27–30 °C. The AuNRs growth was indicated by the gradual color change from colorless to dark blue within ∼30 min.^18^

The mechanisms behind the formation of AuNRs are still a subject of ongoing research. In the seed-mediated protocol, CTAB is a suitable surfactant for AuNRs synthesis because it is both a stabilizer and a structure-directing agent.^23^ CTAB assembles in rod-like micelles in an aqueous solution, which is expected to induce anisotropic growth on spherical particles.^23^ Furthermore, CTA^+^ cations of the CTAB molecules are electrostatically adsorbed to bromide anions bound to AuNR's surface, preventing particles from aggregating or coalescing during the synthesis.^24^ Several groups have proposed a more important role of the bromide counterion in anisotropic growth. However, CTAB remains the most employed surfactant.^25^

#### Synthesis of europium-doped silica host matrices with and without AuNRs

**Fig. 1:**
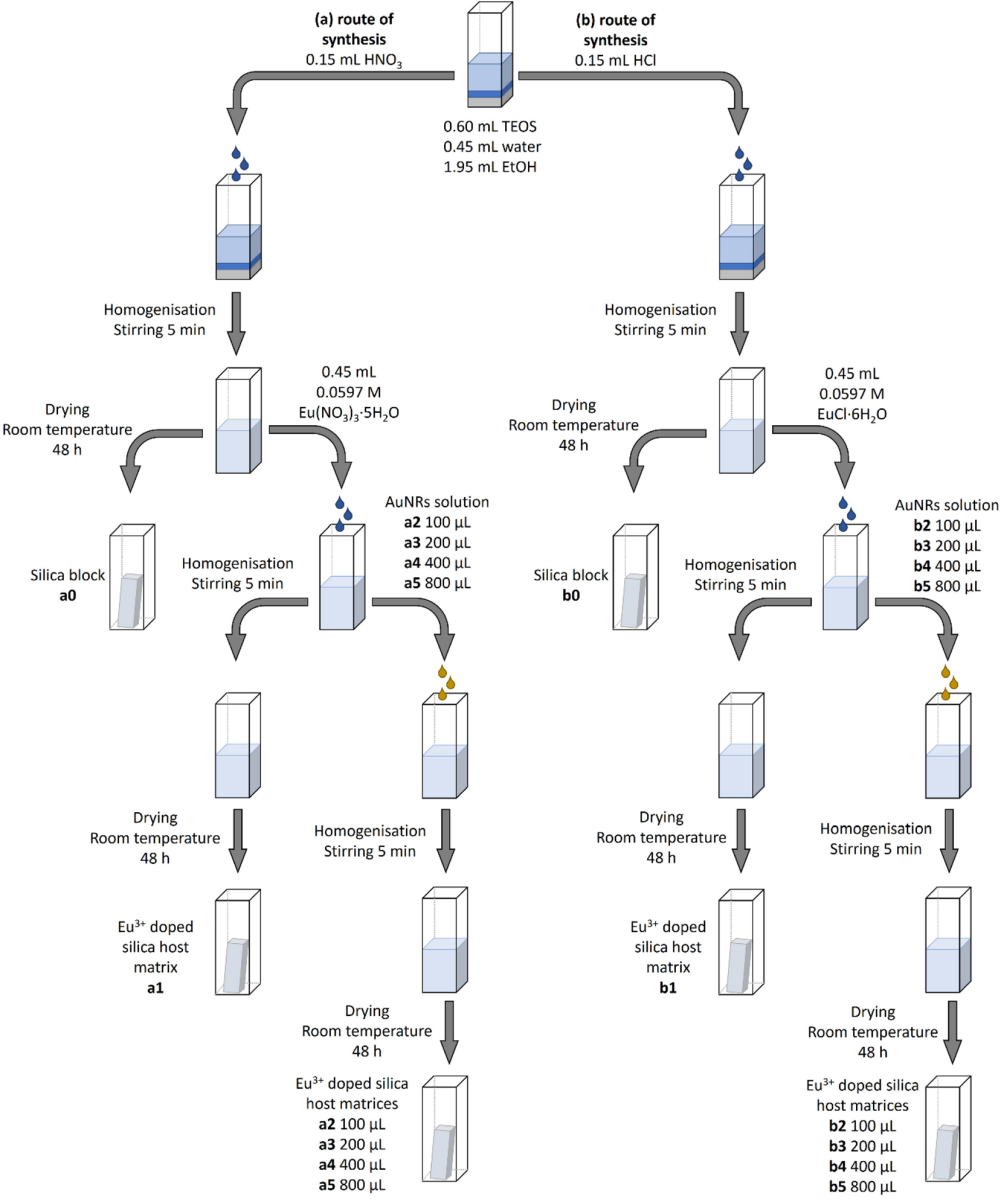
The synthesis routes used to obtain the intended nanostructured composites based on europium-doped silica host matrices.

These materials were prepared using the water–ethanol-TEOS phase diagram,^26^ where TEOS is the precursor, and ethanol is the homogenizing agent. A composition in the miscible area of the phase diagram was selected to obtain transparent glasses: 0.60 mL TEOS, 0.45 mL water and 1.95 mL ethanol. Pure silica host matrices were also prepared using this stoichiometry as the reference but using two different catalysts, route of synthesis (a) and (b) (see Table 1 and Fig. 1).

Europium-doped silica blocks were obtained by adding either 1% M of Eu(NO_3_)_3_·5H_2_O (a1–a5 samples) or EuCl·6H_2_O (b1–b5 samples) to TEOS, respectively (see Table 1). Then, the intended nanostructured composites based on europium-doped silica host matrices were obtained following the same methodology but adding AuNRs suspension at various concentrations as described in Table 1 and Fig. 1.

### Characterization

UV-vis spectra were obtained using a PerkinElmer Lambda 10 spectrophotometer. STEM images were obtained using the JEOL JEM 2200FS+CS microscope. The BSE images were obtained using either the HITACHI SU3500 electron microscope equipped with XMax^N^ 50 SDD EDS for the elemental analysis or the JEOL JSM 7401F. The photoluminescence (PL) characterization was conducted using a Horiba Jobin-Yvon (FL-1005) spectrometer equipped with a 450 W Xe lamp. The PL emission spectra were obtained using 394 nm as excitation wavelength, and the PL excitation spectra were recorded by monitoring the 614 nm emission band. The (*x*,*y*) chromaticity coordinates were calculated from the PL emission spectra of the samples according to The Commission International de I'Eclairage (CIE system of colorimetry 1931).^27^

## Results and discussion

### Gold nanorods (AuNRs)

**Fig. 2:**
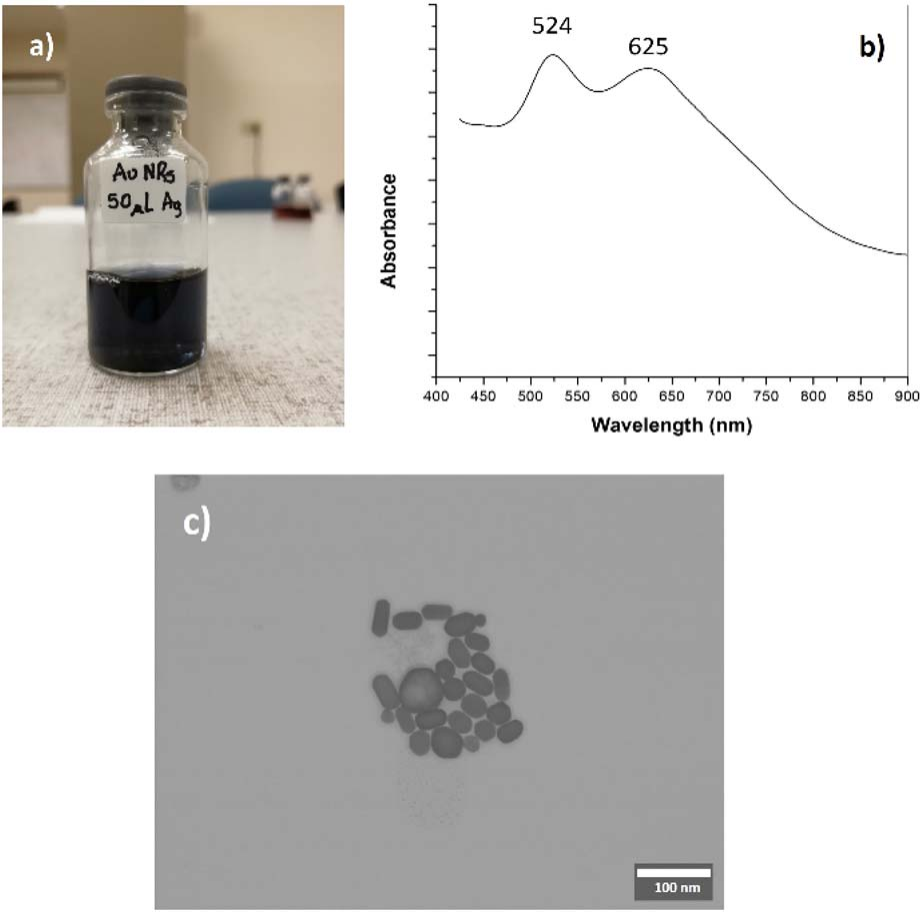
Gold nanorods (AuNRs) obtained by the seeding method [18]. (a) AuNRs suspension; (b) absorption spectrum and (c) STEM image (for better details, the readers are referred to the digital images provided as ESI†).

Fig. 2a shows the AuNRs suspension obtained from the methodology reported in the literature.^18^ Its absorption spectrum comprises two maxima at 524 and 625 nm (see Fig. 2b), typically associated with the transversal and longitudinal plasmons, respectively. According to the literature, these values are consistent with an aspect ratio of 2.15, approximately.^28,29^ STEM images showed that the morphology of the AuNRs includes nanoparticles with different sizes and shapes (see Fig. 2c), but it is evident the presence of nanorods with low aspect ratio. Using the STEM images, we conducted a visual quantification: large 40.8 ± 5.5, width 23.8 ± 3.1 nm, and aspect ratio 1.7.

### Electron microscopy analysis

**Fig. 3:**
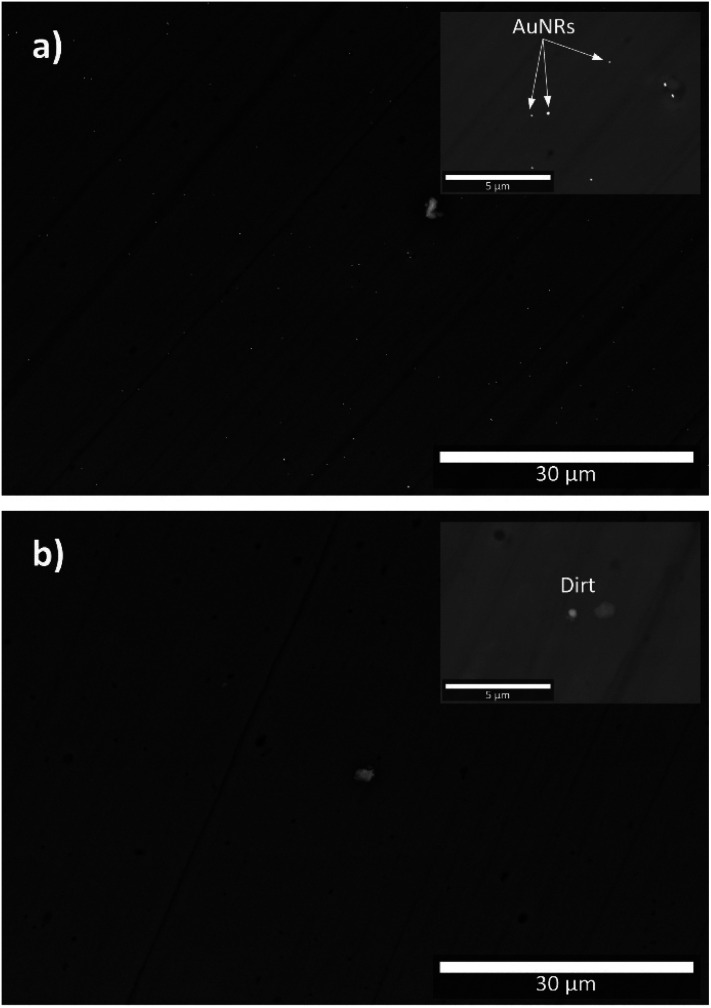
BSE images of: (a) a5 sample; (b) b5 sample. Insets correspond to samples observed at higher magnifications (for better details, the readers are referred to the digital images provided as ESI†).

Fig. 3a and b show the BSE images of the cross-section of a5 and b5 samples, respectively. These samples were selected for the SEM analysis because they were fabricated with the highest volume of AuNRs suspension (see Table 1 and Fig. 1). It is worth mentioning that imaging the samples was difficult because their non-conductive characteristic produced considerable static surface charge. Since the AuNRs were sparsely populated on the surface of the analysis, this feature was another factor that caused difficulties in imaging the samples.

**Fig. 4:**
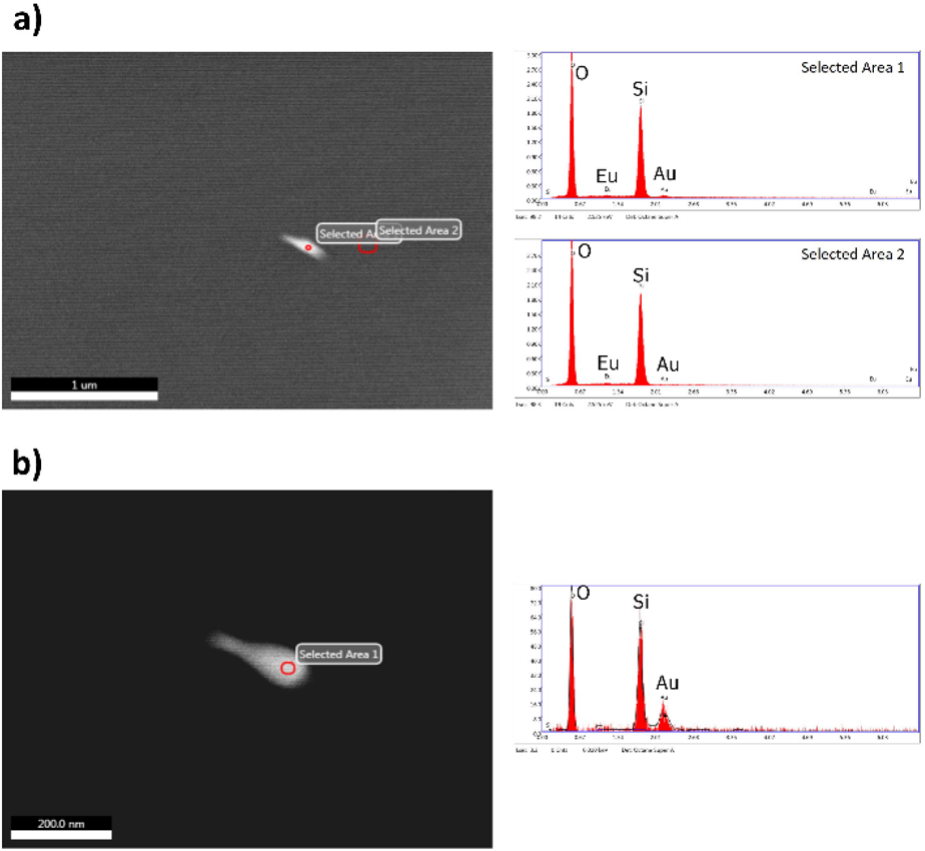
EDS analysis carried out on (a5) sample. In (a) the individual EDS analysis marked as selected area 1 over AuNRs and selected area 2 on the surface of the silica matrix correspond with the spectra to the left. In (b) EDS analysis was carried out at higher magnifications to improve local chemical analysis (for better details, the readers are referred to the digital images provided as ESI†).

The BSE image of a5 shows small bright dots randomly distributed across the sample. Because of their high contrast, this feature can be attributed to the presence of AuNRs embedded in the a5 sample (see Fig. 3a). The inset in Fig. 3a is a BSE image of the cross-section of a5 at higher magnification, which shows in more detail the identified AuNRs. A closer examination at higher magnifications revealed that these bright spots are clusters comprised of a few AuNRs because their size can be up to 200 nm (see Fig. 4a and b), while the size of the AuNRs previously determined was 40.8 ± 5.5 nm large and 23.8 ± 3.1 nm wide (see Fig. 2).

To estimate AuNRs population density in the cross-section of the a5 sample, we measured the number of bright spots per 204 μm^2^ (see Fig. S1†). For this purpose, we divided the micrograph shown in Fig. 3a into 25 rectangles of approximately 12 μm wide per 17 μm large (see Fig. S1†). Also, the following assumption was considered: an isolated bright spot is defined when it is notably separated from others, independently if it is a cluster of AuNRs. The results showed 5 ± 2 bright spots per 204 μm^2^ on average, indicating how the AuNRs are sparsely distributed on the a5 sample. Fig. S2† shows the results obtained in the a4 sample for comparison.

Fig. 4 shows the EDS analyses carried out in areas of different contrast on the a5 sample. The EDS analysis in the area with bright contrast indicated the presence of gold, but the signal was weak due to the small volume of the AuNRs compared to the host. Other important features that complicated the EDS analysis were that the AuNRs are embedded in the silica host and the non-conductive characteristic of the samples. Fig. 4b shows the EDS analysis of the same particle at higher magnifications to improve the chemical analysis.

SEM analysis confirmed the presence of AuNRs randomly distributed in samples obtained through the (a) synthesis route. In this case, the chemicals used during the sol–gel process did not have a negative impact on the stability of the AuNRs. On the other hand, no AuNRs were observed in the samples obtained through the (b) route of synthesis (see Fig. 3b). Thus, the use of EuCl·6H_2_O and HCl was not favorable to the stability of AuNRs, which is in good agreement with the literature.^30,31^

### Luminescence

#### Emission spectra

**Fig. 5:**
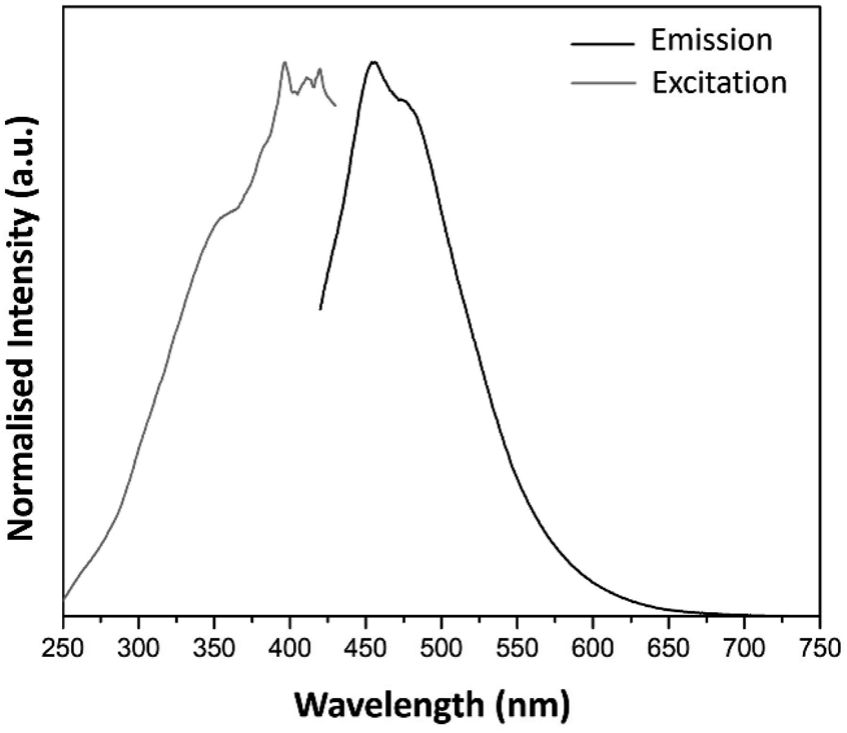
Excitation (*λ*_em_ = 453 nm) and emission spectra (*λ*_ex_ = 394 nm) of pure SiO_2_ host matrix obtained by sol–gel technique.

Fig. 5 shows the PL spectra of the pure silica host matrix as a reference. Upon excitation with 394 nm, the sample showed intense blue luminescence, evidenced by the PL emission spectrum as a broad band with maxima located at 453 and 475 nm (see Fig. 5). This band extends from 420 to 700 nm. A literature review suggested that this feature results from oxygen-related intrinsic defects.^32^Fig. 5 also shows the excitation spectrum of the host monitoring the 453 nm emission band.

**Fig. 6:**
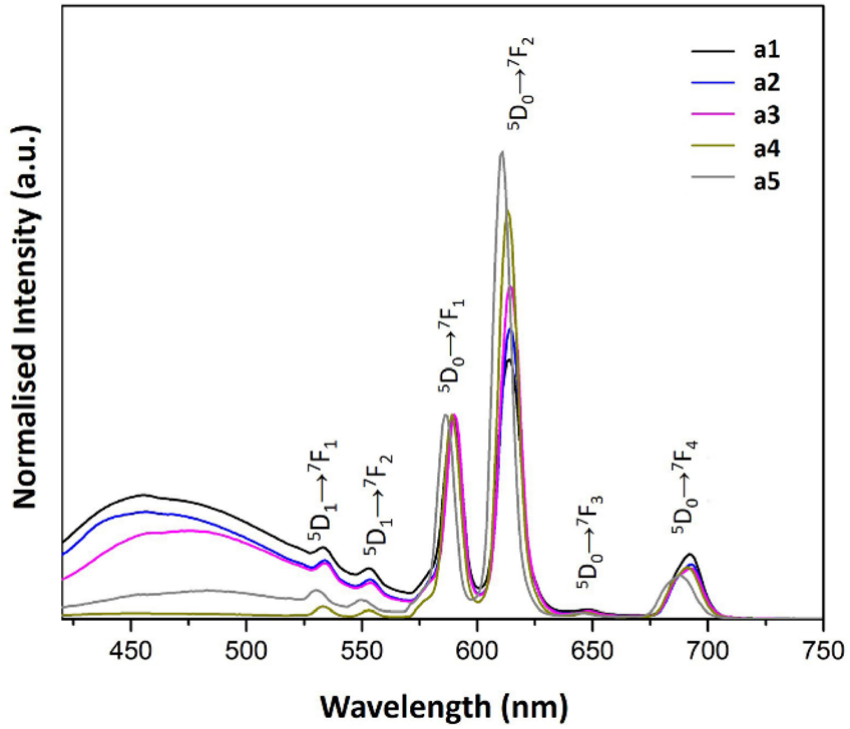
PL emission spectra of a1–a5 samples prepared with AuNRs suspension at various concentrations (*λ*_ex_ = 394 nm).

**Fig. 7:**
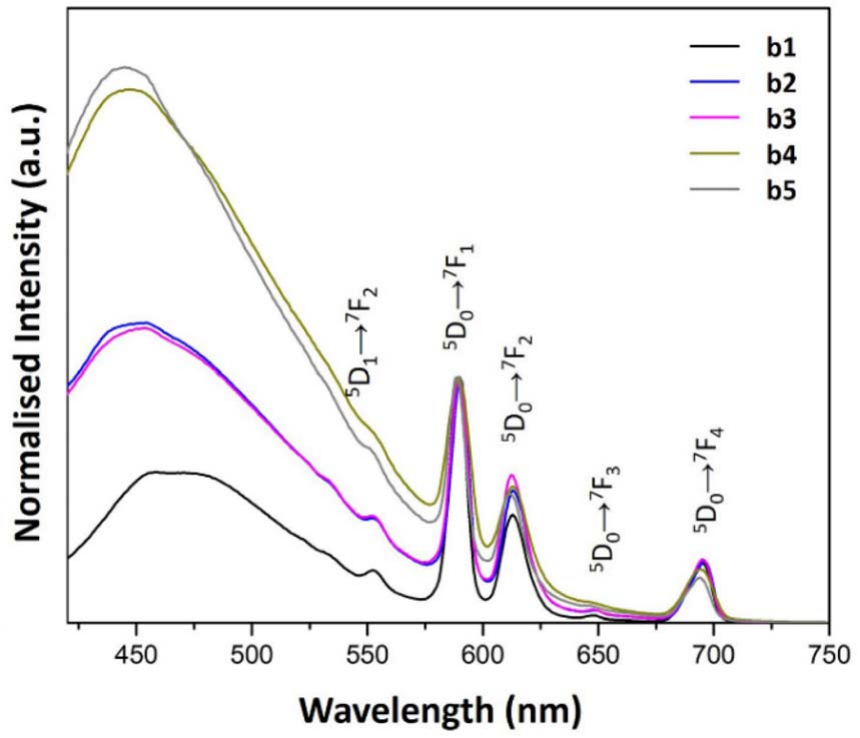
PL emission spectra of b1–b5 samples prepared with AuNRs suspension at various concentrations (*λ*_ex_ = 394 nm).

The PL emission spectra of a1–a5 and b1–b5 samples are shown in Fig. 6 and 7, respectively. The spectra were obtained upon excitation of the ^7^L_6_ state of Eu^3+^. Hence, 394 nm was selected because the PL excitation spectra of the samples suggested that this wavelength is effective when monitoring the hypersensitive ^5^D_0_ → ^7^F_2_ emission band of Eu^3+^.

Besides, the analysis of the Eu^3+^ contribution to these spectra was conducted according to the following: the PL emission spectrum of the Eu^3+^ ion shows two distinctive bands associated with the ^5^D_0_ → ^7^F_1_ and the ^5^D_0_ → ^7^F_2_ transitions. While the former is not affected by the Eu^3+^ local symmetry, the latter is recognized as a hypersensitive transition greatly affected by the local symmetry and electric field around the Eu^3+^.^14,33^ According to the above arguments, we have normalized the emission intensities of the PL bands involving Eu^3+^ transitions, considering the ^5^D_0_ → ^7^F_1_ as a reference.^14^

Also, we can use the relative intensity ratio (^5^D_0_ → ^7^F_2_/^5^D_0_ → ^7^F_1_) as an indicator to determine whether the local symmetry of the Eu^3+^ ions is centrosymmetric or non-centrosymmetric. For this purpose, we calculated the integrated areas under the curves of the ^5^D_0_ → ^7^F_2_ and ^5^D_0_ → ^7^F_1_ bands to obtain the relative intensity ratio (see Table 2).

**Table:** The relative intensity ratio ^5^D_0_ → ^7^F_2_/^5^D_0_ → ^7^F_1_ of the samples

Sample	^5^D_0_ → ^7^F_2_/^5^D_0_ → ^7^F_1_ intensity ratio
a1	1.61
a2	1.77
a3	2.04
a4	2.34
a5	2.28
b1	0.56
b2	0.70
b3	0.78
b4	0.62
b5	0.72

Regarding a1–a5 samples, the ^5^D_0_ → ^7^F_2_/^5^D_0_ → ^7^F_1_ relative intensity ratio shown in Table 2 suggested that the Eu^3+^ ions have a non-centrosymmetric environment. Furthermore, there is an enhancement in the intensity of the Eu^3+^ luminescence as the AuNRs concentration increases (see Fig. 6 and Table 2). In other words, adding AuNRs enhances the contribution of the Eu^3+^ ions to the overall luminescence of these samples. This characteristic gives the distinctive reddish-orange color emitted from the samples.

Another feature observed in the spectra of the a1–a5 samples is the presence of bands associated with transitions from a higher excited state of Eu^3+^, ^5^D_1_. This feature is more commonly observed in inorganic host lattices than in organic materials because, in molecular-based materials, non-radiative transitions are more favorable due to the degree of freedom of molecular species.^14^

The PL emission spectra of b1–b5 samples also showed the characteristic bands of the Eu^3+^ ion. Hence, apart from the PL band owned by the SiO_2_ matrix, the ^5^D_0_ → ^7^F_1_ transition was the most intense band. Thus, the contribution of the ^5^D_0_ → ^7^F_2_ band, which is recognized as a hypersensitive transition, was affected by the local symmetry of the Eu^3+^.^14^ According to Table 2, it is suggested that the dominant local environment of the Eu^3+^ ion in b1–b5 samples is centrosymmetric. Hence, the luminescent characteristics of Eu^3+^ resemble those found in water.^34^ This property is attributed to the presence of water and ethanol trapped in liquid form inside the host matrix since the samples were dried at room temperature. To remove solvent molecules more firmly attached to the host matrix, heating the samples at higher temperatures is necessary, but it is beyond the present study.

Table 2 also shows that the concentration of AuNRs suspension has little impact on the ^5^D_0_ → ^7^F_2_/^5^D_0_ → ^7^F_1_ relative intensity ratio in the b1–b5 samples, suggesting that adding AuNRs suspension did not cause significant changes in the local environment of Eu^3+^ ions.

On the other hand, the addition of AuNRs suspension in samples b1–b5 produced a blue shift of the host matrix emissions. Furthermore, increasing the concentration of AuNRs suspension enhanced the luminescence of the host matrix. Consequently, as the AuNRs suspension concentration increases, the band from 420–550 nm contributes significantly to the overall luminescence of the samples (see Fig. 7). This feature produced an intense blue contribution to the total emission, giving the blue variations of the b1–b5 samples.

Samples b2 and b3 produced similar emission spectra (see Fig. 7). Nevertheless, there is a dependence on the intensity of the blue contribution in b4 and b5. These results suggested a discrete optical contribution to the overall luminescence of the samples for higher concentrations of AuNRs suspension.

The blue luminescence of the host matrix associated with oxygen defects was previously studied in detail.^35^ Hence, the characteristic SiO_2_ band around 459 nm was determined to be the result of defect luminescence caused by two-fold coordinated silicon centers due to oxygen deficit.^36^ In the present study, the complexity of the chemical mix during the sol–gel process may affect both the intensity and shift of the band due to the changes in the network that precursors and other chemicals induced during synthesis. In this regard, these substances could react to form complexes, which produce changes in the network and consequently alter the luminescent characteristics of the band. The study of the mechanisms involved in the changes observed in the luminescence of the matrix with the increase of AuNRs suspension is beyond the present work.

In addition, the enhancement in the intrinsic luminescence of the host matrix suggests that charge transfer may also occur between gold species and oxygen defects in the b1–b5 samples.^16^

From the above results, it is clear that the Eu^3+^ local environment is significantly affected by the europium salt and catalyst employed in the synthesis route. Furthermore, in both synthesis routes, the presence of Au influences the PL spectra, displaying concentration-dependent characteristics. Since BSE confirmed the presence of stable AuNRs in a1–a5 samples, the changes in the luminescent characteristics could be associated with the contribution of the LSPR of AuNRs in the vicinity of Eu^3+^ ions. On the other hand, the subtle increase in the ^5^D_0_ → ^7^F_2_ band found in the b1–b5 samples, where no AuNRs were observed, could be attributed to energy transfer between non-plasmonic gold species, *e.g.*, small AuNPs, clusters and molecule-like gold particles, with Eu^3+^ ions.^16^

#### Excitation spectra

**Fig. 8:**
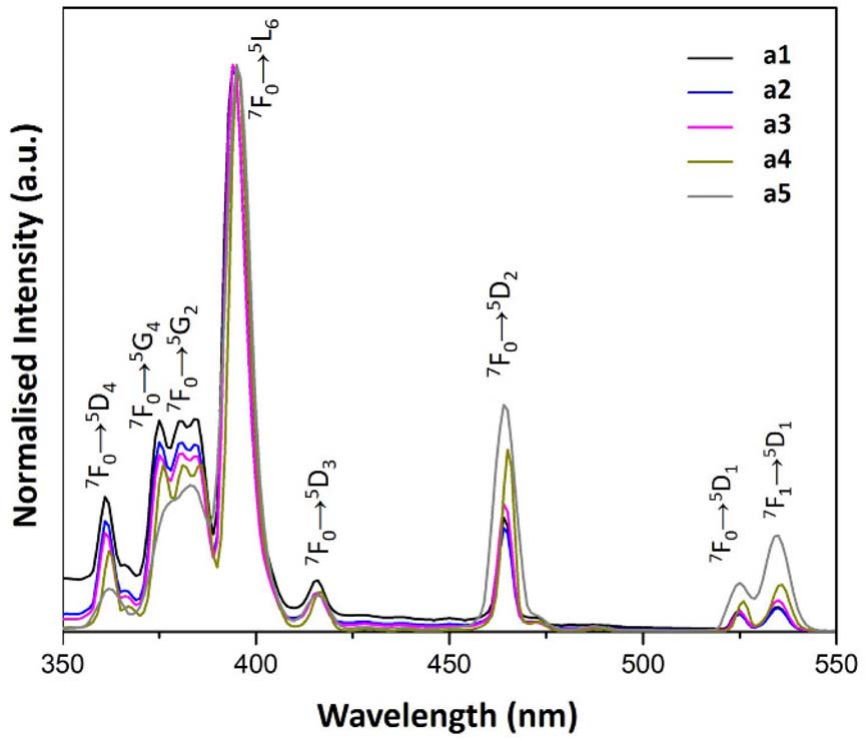
PL excitation spectra of a1–a5 samples prepared with AuNRs suspension at various concentrations (*λ*_em_ = 614 nm).

**Fig. 9:**
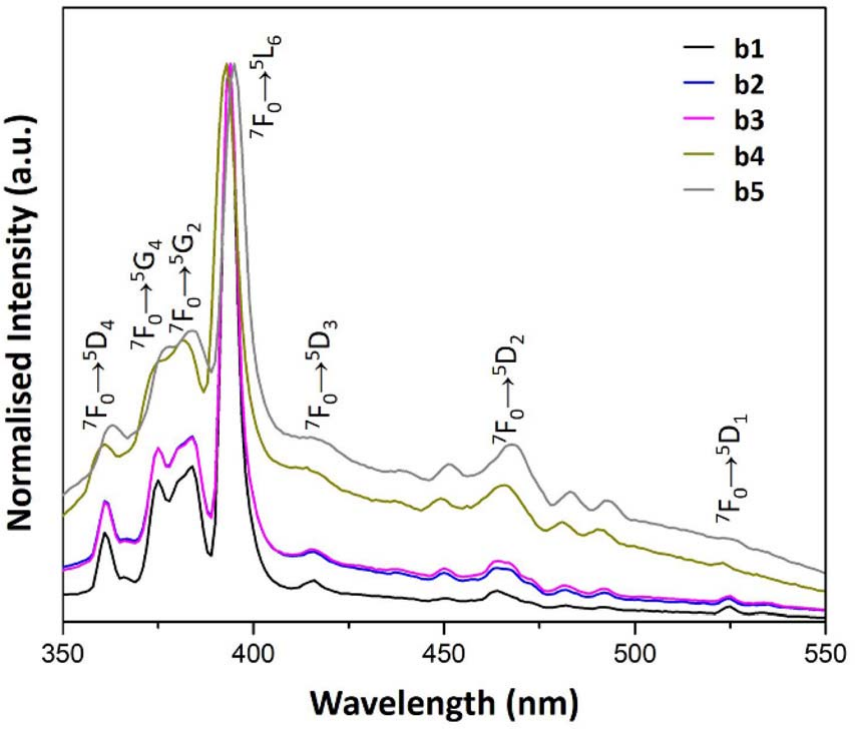
PL excitation spectra of b1–b5 samples prepared with AuNRs suspension at various concentrations (*λ*_em_ = 614 nm).

According to the literature, LSPR and energy transfer affect both the emission and the PL spectra.^37^Fig. 8 and 9 show the PL excitation spectra of a1–a5 and b1–b5 samples, respectively. In all the spectra, the most intense band corresponds to the ^7^F_0_ → ^5^L_6_ transition, a notable characteristic of the PL excitation spectra of Eu^3+^.

The PL excitation spectra of a1–a5 samples showed sharp bands as the result of the electronic transitions of the Eu^3+^ ions. Besides, the characteristic transitions ^7^F_1_ → ^5^D_1_, ^7^F_0_ → ^5^D_1_ and ^7^F_0_ → ^5^D_2_ with maxima at 540, 530 and 470 nm enhance their intensity with AuNRs suspension concentration. This property could be responsible for the enhancement of Eu^3+^ luminescence in these samples because there is an overlap of the ^7^F_1_ → ^5^D_1_ and ^7^F_0_ → ^5^D_1_ bands with the LSPR bands of AuNRs (see Fig. 5 and 8). Therefore, the AuNRs sensitize the Eu^3+^ ions, enhancing their luminescence as observed in the corresponding emission spectra (see Fig. 6). On the other hand, the ^7^F_1_ → ^5^D_2_ band does not overlap with the LSPR window, but due to its hypersensitive nature, its intensity enhances with the increase of AuNRs concentration.

In contrast, the ^7^F_1_ → ^5^D_1_, ^7^F_0_ → ^5^D_1_ and ^7^F_0_ → ^5^D_2_ bands in the PL excitation spectra of the b1–b5 samples are not well-defined. The broadening of spectral lines observed in b1–b5 samples compared to a1–a5 samples results from the difference in the chemical environment of Eu^3+^ ions. The apparent increase in the excitation window between 350 and 550 nm can be seen as a result of the quenching of the ^7^F_0_ → ^5^L_6_ band. This feature could be responsible for the reduced contribution of the Eu^3+^ ions to the overall luminescence of these samples when increasing the concentration of AuNRs suspension.

#### Proposed PL mechanism in a2–a5 samples

**Fig. 10:**
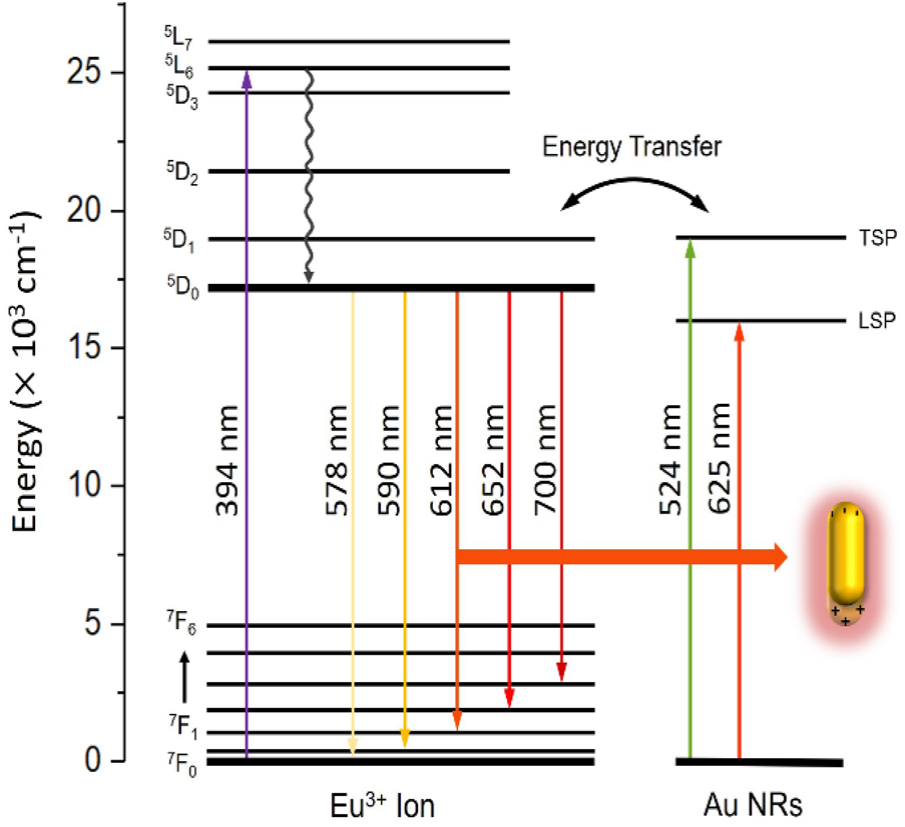
Proposed energy level diagram of Eu^3+^ ions in the vicinity of AuNRs showing the mechanisms for enhanced emission coupling in a1–a5 samples.

Fig. 10 shows the proposed mechanism when Eu^3+^ ions are near AuNRs. The above results show that the Eu^3+^ local environment is significantly affected by using different chemicals as dopants and catalysts.

In the vicinity of plasmonic AuNRs, Eu^3+^ ions can be more efficiently activated from the contribution of the LSPR, inducing a concentrated electric field. These intense local fields increased excitation at 450–550 nm observed in samples a2–a5 (see Fig. 8 and 10). It is worth mentioning that the excitation spectra of samples containing both Eu^3+^ ions and AuNRs do not show NP absorption characteristics. Therefore, energy transfer from excited AuNRs to Eu^3+^ ions is not likely to occur in these systems.^38,39^

Nonetheless, there is another effect that is potentially more important. AuNRs plasmonic bands overlap with the emission wavelengths corresponding to the ^5^D_0_–^7^F_j_ transitions of Eu^3+^ ions. In these conditions, an excited Eu^3+^ ion can interact with a nearby AuNR to induce a plasmon, which then can decay non-radiatively or re-radiate to the far field and create observable emission^40^ (see Fig. 10). Due to the combined nature of the emission, whose spectra remain the same as the fluorophore, the emission is said to come from a fluorophore-metal complex.^41^ At 394 nm excitation, it can be found that AuNRs mainly influence the emission process rather than the excitation process, with the hypersensitive ^5^D_0_ → ^7^F_2_ transition being the most affected.

It is apparent from a1–a5 samples that the PL emission shows a clear dependence on NP concentration. As the concentration increases, particles become closer to each other, so efficient plasmon coupling enhances the emission of Eu^3+^ and AuNRs species. It should be noticed that the influence of Eu^3+^ bands on the overall emission of a1–a5 samples, when compared to the silica band centered at 400 nm, reaches a maximum for sample a4. We attribute this effect to quenching for the sample with the highest concentration of AuNRs (a5), as the intensity of LSPR drops at a critical aggregation of NRs,^42^ reducing the emission of the Eu^3+^-AuNRs species.

### CIE data

**Fig. 11:**
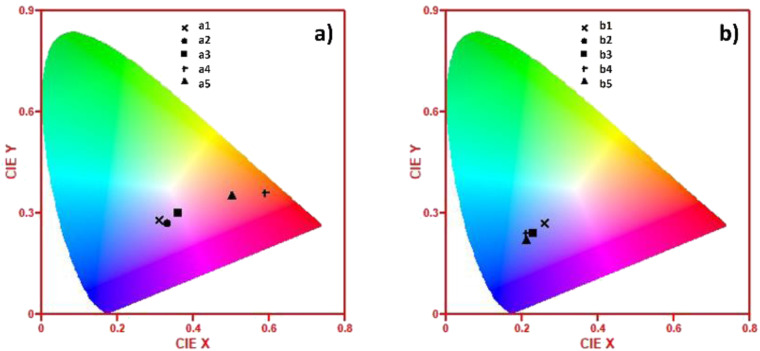
CIE 1931 chromaticity diagrams showing the (*x*,*y*) coordinates of: (a) a1–a5 samples and (b) b1–b5 samples.

Fig. 11 shows the (*x*,*y*) chromaticity coordinates of the a1–a5 and b1–b5 samples plotted in CIE (1931) diagrams to display the correlated color temperature (CCT). In the case of a1–a5 samples, AuNRs produced more significant changes in the CCT. Hence, the color of the light emitted for the sample without AuNRs is also located in the white region of the CIE (1931) diagram. However, as the concentration of AuNRs increases, the color of the samples is remarkably tuned towards the reddish-orange region. This feature is attributed to the contributions of the LSPR, and it can be further exploited for designing novel materials with tunable optical properties for biotechnology, medicine, energy conversion, and lighting. However, sample a5 surpasses the critical aggregation of NRs, reducing the emission of the Eu^3+^ and AuNRs species and resulting in a blue-shift of the color emission.

Conversely, in the b1–b5 samples, the low intensity of the Eu^3+^ luminescent bands, due to its local symmetry and the null contribution of AuNRs, produced light emitting in the white region in the CIE (1931) diagram. Hence, the concentration of Au species quenched the Eu^3+^-PL contribution and tuned the color of the samples towards the blue region.

## Conclusions

The methodology described in this work helped to study the effects of the chemicals used for the fabrication *via* the sol–gel technique of nanostructured composite materials comprising AuNRs. In this regard, the BSE and EDS results suggested that structurally stable AuNRs on the silica host matrices highly depend on the chemicals used to synthesize these materials. In this regard, the synthesis route (a), which involved the use of Eu(NO_3_)_3_·5H_2_O and HNO_3_ as a dopant and catalyst, respectively, not only favored a better stabilization of the AuNRs but also enhanced the luminescent intensity of the Eu^3+^ ions. This feature was further strengthened by increasing the AuNRs suspension concentration in the a1–a4 samples as a result of the coupling between the emission and excitation luminescent bands of Eu^3+^ with the SPR of AuNRs. Hence, the hypersensitive transitions contributed significantly to the overall luminescence of the a1–a5 samples, tunning the color of the light emitted towards the reddish-orange region in the CIE (1931) diagram because of the intense contribution of Eu^3+^ ion to the overall luminescence of the sample. Hence, the activation of the ^5^D_0_ → ^7^F_2_ transition played an important role. On the other hand, chemicals such as EuCl·6H_2_O and HCl, used for the synthesis of b1–b5 samples, dissolved the AuNRs. These findings can help to set the basis for designing novel materials with tunable optical properties with applications in biotechnology, medicine, energy conversion, and lighting.

## Author contributions

J. R. Montes-Bojorquez: carried out experimental work (synthesis and PL experiments), analyzed, organized, and interpreted data. O. Hernández-Negrete: planned the work and strategies, analyzed data, interpreted data (microscopy), discussed results, and wrote the draft. H. E. Esparza-Ponce: planned and carried out experimental work (microscopy) and analyzed data. V. Alvarez-Montaño: analyzed data, planned strategies, and discussed results. J. Hernández-Paredes: conceived the idea, supervised the work, planned strategies, interpreted data, discussed results, and wrote the draft.

## Conflicts of interest

There are no conflicts to declare.

## Supplementary Material

RA-013-D3RA04652D-s001

RA-013-D3RA04652D-s002

RA-013-D3RA04652D-s003

RA-013-D3RA04652D-s004

RA-013-D3RA04652D-s005
