# Supplementary material for: Sol–gel synthesis of Eu3+ doped silica-gold nanorod composites with tunable optical properties
Source: RSC Adv. 2023 Sep 8;13(38):27006–15. doi: 10.1039/d3ra04652d (PMC10485736; doi:10.1039/d3ra04652d)
Supplement: RA-013-D3RA04652D-s001 [file RA-013-D3RA04652D-s001.pdf]

Electronic Supplementary Material (ESI) for RSC Advances.
This journal is © The Royal Society of Chemistry 2023

Electronic Supplementary Information

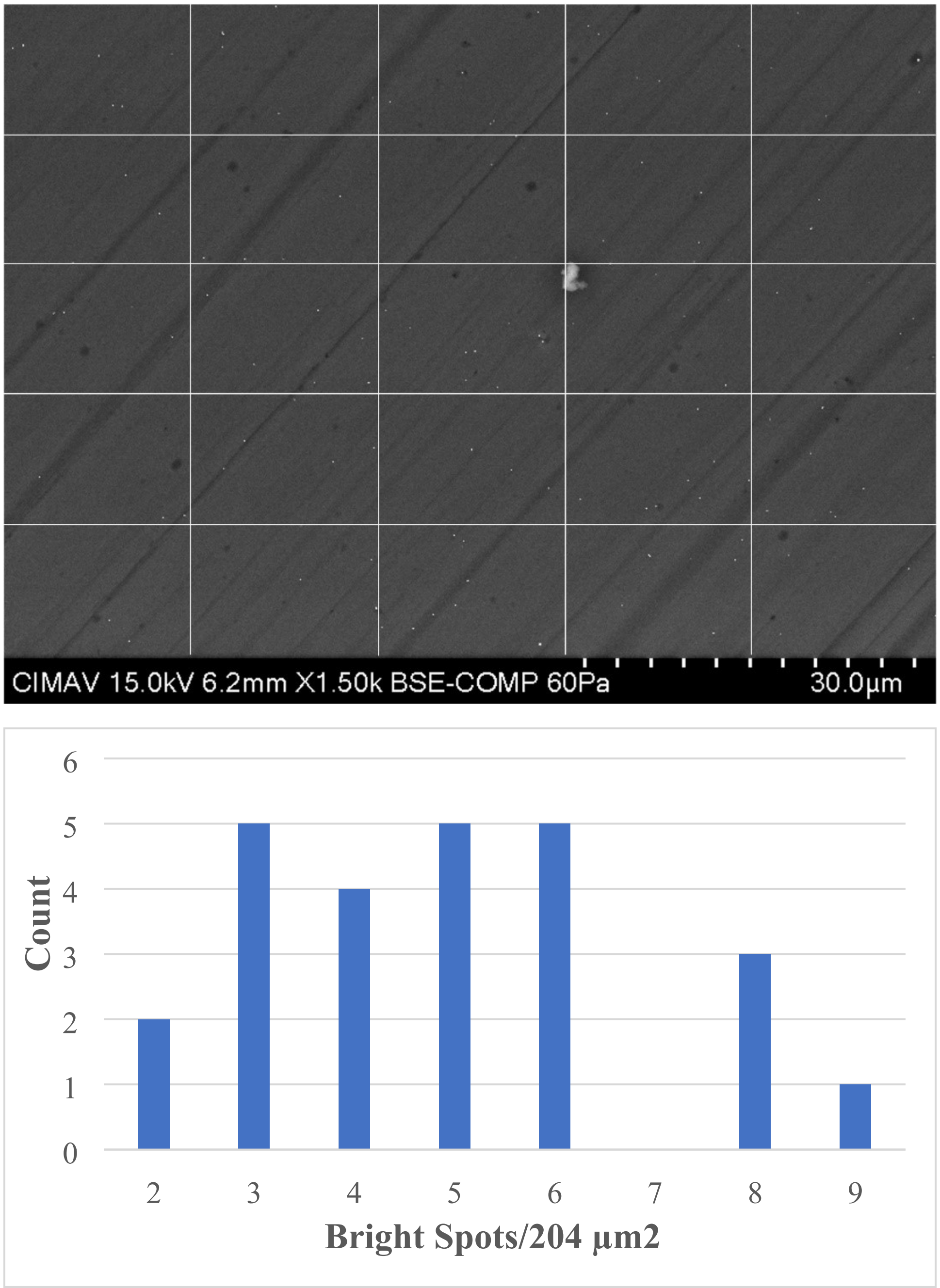

Fig. S1. Analysis of AuNRs randomly distributed in **a5** sample.

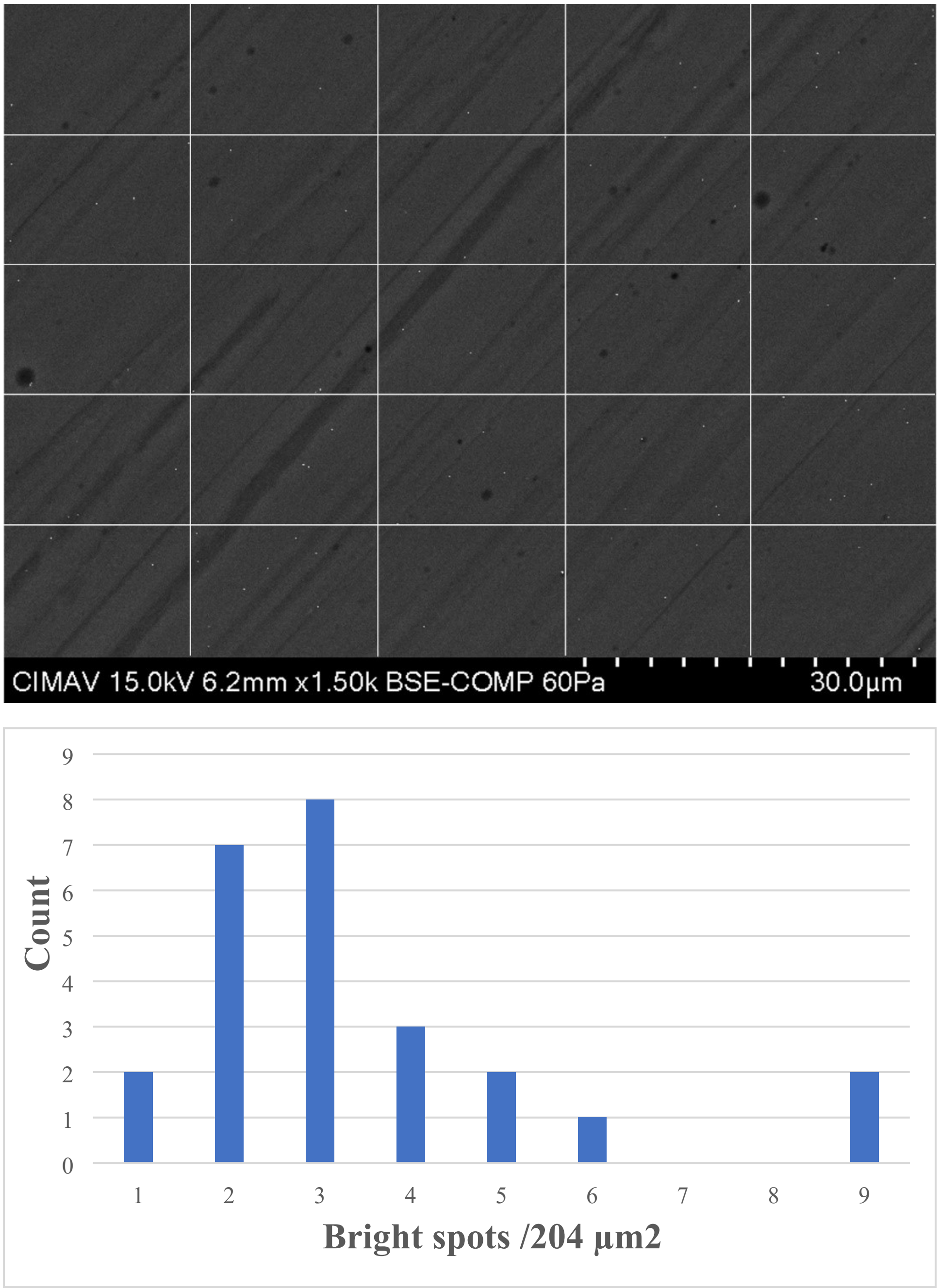

Fig. S2. Analysis of AuNRs randomly distributed in **a4** sample.

Electron Microscopy Images

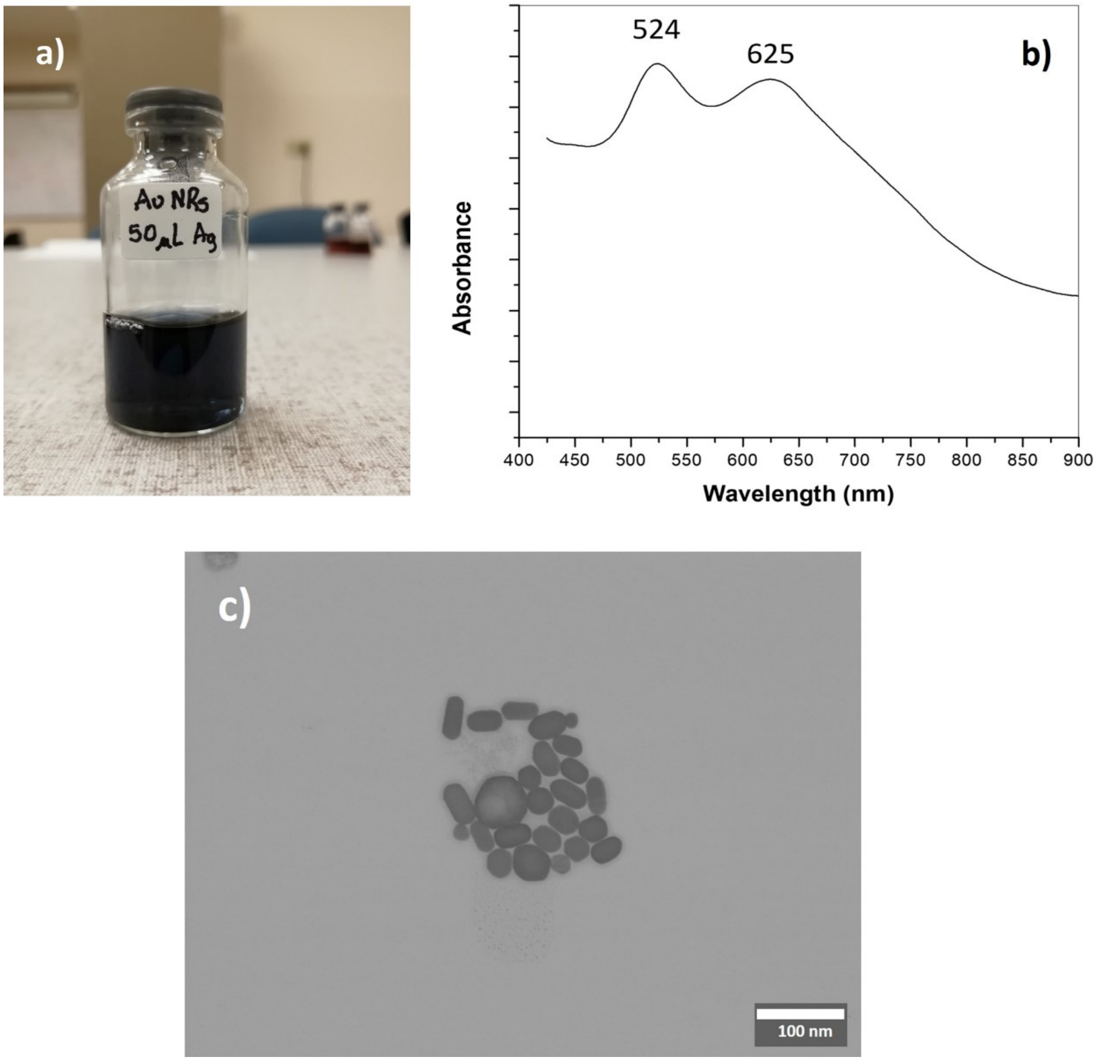

Fig. S3 Gold nanorods (AuNRs) obtained by the seeding method [18]. a) AuNRs suspension; b) absorption spectrum and c) STEM image.

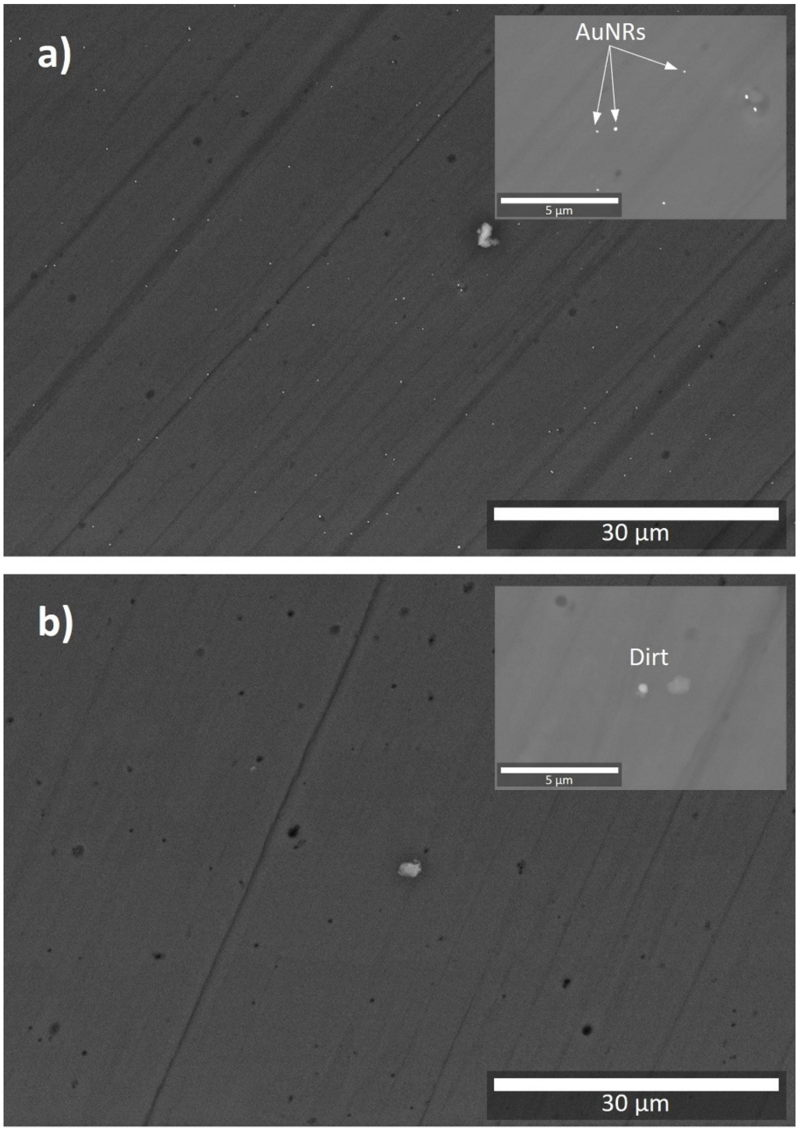

Fig. S4 BSE images of: a) **a5** sample; b) **b5** sample. Insets correspond to samples observed at higher magnifications.

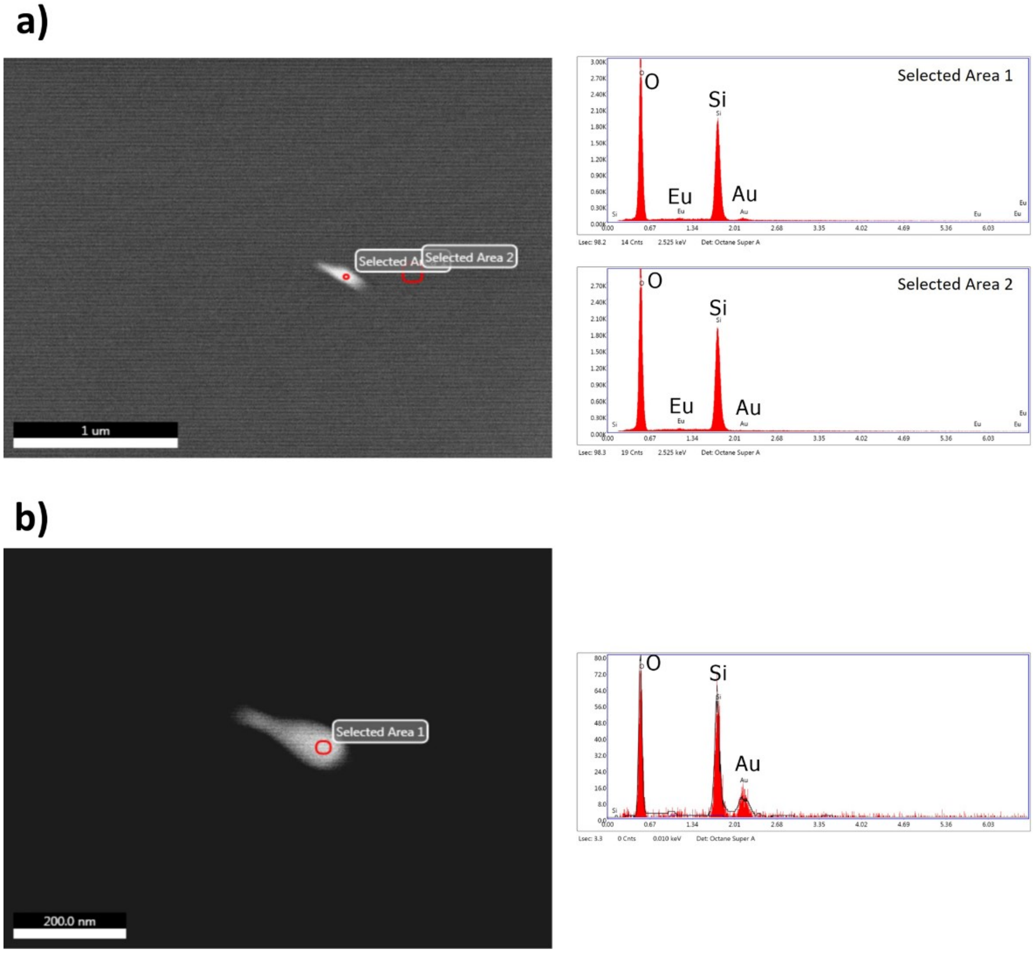

Fig. S5 EDS analysis carried out on **(a5)** sample. In a) the individual EDS analysis marked as Selected Area 1 over AuNRs and Selected Area 2 on the surface of the silica matrix correspond with the spectra to the left. In b) EDS analysis was carried out at higher magnifications to improve local chemical analysis.
