# Supplementary figures and images for: Sol–gel synthesis of Eu3+ doped silica-gold nanorod composites with tunable optical properties
Source: RSC Adv. 2023 Sep 8;13(38):27006–15. doi: 10.1039/d3ra04652d (PMC10485736; doi:10.1039/d3ra04652d)

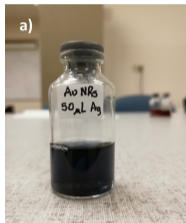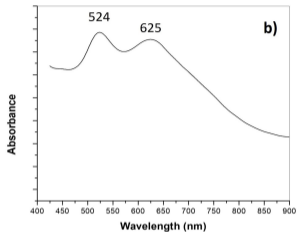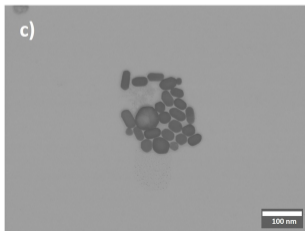

Supplement: RA-013-D3RA04652D-s003 [file RA-013-D3RA04652D-s003.pdf]

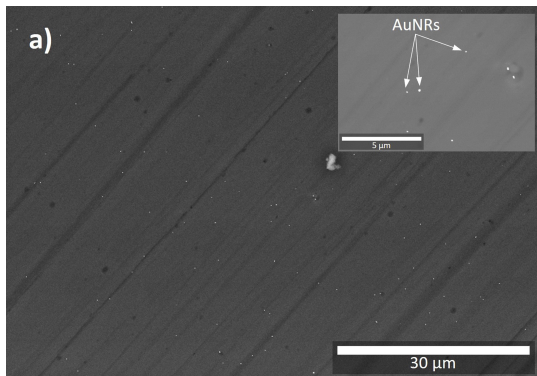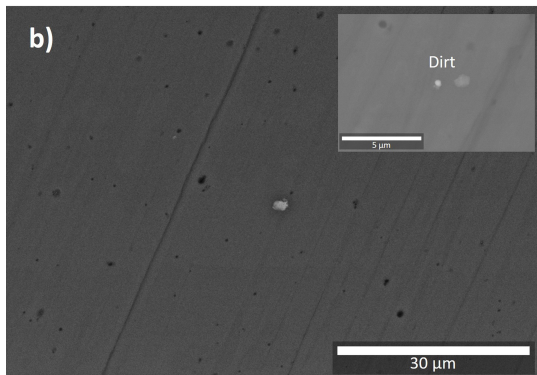

Supplement: RA-013-D3RA04652D-s004 [file RA-013-D3RA04652D-s004.pdf]

a)

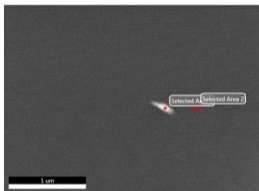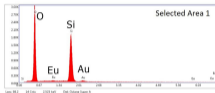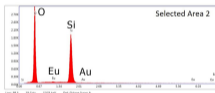

b)

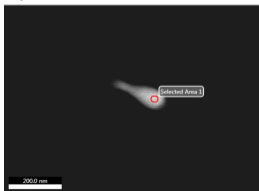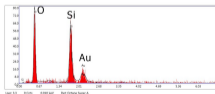

Supplement: RA-013-D3RA04652D-s005 [file RA-013-D3RA04652D-s005.pdf]
